# The effect of Chlorohexidine, Er:YAG laser and diode laser 980 nm as dental cavity disinfectants on dentine morphology and microleakage of composite restoration: an in vitro study

**DOI:** 10.1038/s41405-025-00391-z

**Published:** 2026-01-15

**Authors:** Mennatallah Khafagi, Mostafa Gheith, Haythem S. Moharrum, Mohamed Abo Elyazeed Ahmed, Riham M. Aly, Maryam El Mansy

**Affiliations:** 1 Research Assistant – Pediatric Dentistry Orthodontics and Pediatric Dentistry Department, Oral and Dental Research Institute, National Research Centre, Dokki, Giza Governorate Egypt; 2 Professor of Laser applications in dental medicine, National Institute of Laser Enhanced Sciences, Cairo University, Giza, Egypt; 3 Assistant professor Department of Medical Applications of Laser, National Institute of Laser Enhanced Sciences, Cairo University, Giza, Egypt; 4 Professor of Pediatric Dentistry Orthodontics and Pediatric Dentistry Department, Oral and Dental Research Institute, National Research Centre, Dokki, Giza Governorate Egypt; 5 Stem Cell Laboratory, Center of Excellence for Advanced Sciences, National Research Centre, Cairo, Egypt, Department of Basic Dental Science, Oral Medicine & Dentistry Research Institute, National Research Centre, Dokki, Cairo Egypt; 6 Researcher of Pediatric Dentistry, Orthodontics and Pediatric Dentistry Department, Oral and Dental Research Institute, National Research Centre, Dokki, Giza Governorate 12622 Egypt

**Keywords:** Dentistry, Paediatric dentistry

## Abstract

**Introduction:**

Residual microorganisms may remain even after thorough mechanical cavity preparation, leading to secondary caries. Additionally, the smear layer generated during this process can impair the adhesion between composite resin and dentine and limit the penetration of disinfectants into dentinal tubules. This study aimed to compare the effects of 2% chlorhexidine (CHX), 980 nm diode laser, and Er:YAG laser as cavity disinfectants on dentine morphology, mineral content, and microleakage of composite restorations.

**Materials and Methods:**

Forty extracted sound human primary molars were randomly assigned to four groups (*n* = 10): Group I (negative control, no disinfection), Group II (2% CHX application), Group III (980 nm diode laser, 1 W, continuous wave), and Group IV (Er:YAG laser, 1.2 W). Dentine morphology and restoration microleakage were examined via Scanning Electron Microscopy: (SEM), while mineral content was evaluated using Energy-Dispersive X-ray Spectroscopy (EDS).

**Results:**

SEM analysis showed that CHX (Group II) left smear layer residues with narrowed tubules, while the diode laser (Group III) partially removed the smear layer. Er:YAG laser (Group IV) resulted in complete smear layer removal, wider tubules. EDS revealed significantly higher mineral content (Ca, P and Ca/P ratio)in Group IV compared to Groups II and group III (*p* < 0.001), with no significant difference between Groups II and III. Microleakage was highest in the control group and lowest in the Er:YAG group (*p* < 0.001).

**Conclusion:**

The Er:YAG laser showed enhanced outcomes in improving dentine morphology, increasing mineral content, and minimizing microleakage, making it the most effective disinfectant tested.

## Introduction

Dental caries is a highly prevalent condition worldwide, representing a multifactorial disease that arises from the complex interplay between cariogenic bacteria, dietary sugars, and host-related factors. These microorganisms metabolise dietary sugars, producing acids that progressively demineralise the hard tissues of the tooth, ultimately leading to tooth decay [1].

Cervical caries is a common form of tooth decay, often associated with poor oral hygiene and a highly cariogenic diet. Residual microorganisms may persist even after thorough mechanical preparation, particularly in cases where caries removal is incomplete [2].

These bacteria may penetrate the restoration–tooth interface, promoting microleakage and increasing the risk of secondary, or recurrent, caries. To minimise this risk, disinfection of the dentine surface is recommended following cavity preparation and prior to the placement of any restorative material [3].

Additionally, the smear layer formed during cavity preparation may act as a barrier, preventing adequate bonding between the composite resin restoration and dentine, as well as hindering the effective penetration of disinfectants into the dentinal tubules [2].

Dentine, a mineralised tissue, is composed of both organic and inorganic components. Its inorganic fraction primarily consists of hydroxyapatite crystals containing calcium (Ca) and phosphorus (P). The typical calcium-to-phosphorus (Ca/P) ratio in dentine hydroxyapatite is approximately 1.67, reflecting a stable and consistent mineral composition. However, this ratio may vary depending on the type of crystal, calcium availability, anatomical location, and the method of measurement [4].

Chemical agents used during dental procedures may alter the Ca/P ratio, leading to changes in the structural and chemical integrity of dentine, including its permeability and solubility. This disruption can, in turn, affect the balance between dentine’s organic and inorganic components and negatively impact its physical properties, such as microhardness and surface roughness [5].

Furthermore, these chemical agents may reduce the mineral content of dentine, potentially compromising its mechanical strength and directly affecting the bond between dentine and the restorative material. This, in turn, may influence the long-term durability of the restoration [6].

Microleakage, defined as the undetectable seepage of fluids, bacteria, or ions between the tooth structure and the restoration, is a leading cause of clinical failure [7]. It may result in tooth discolouration, postoperative sensitivity, recurrent decay, and pulpal complications. Therefore, optimising disinfection protocols and enhancing adhesive bonding are essential for reducing bacterial contamination, improving marginal integrity, and ensuring the longevity of composite restorations [8].

Chlorhexidine (CHX) is widely regarded as the gold standard for cavity disinfection due to its potent antimicrobial properties and its ability to bind to bacterial amino acids. Studies have demonstrated that concentrations of up to 10% are considered safe for use on living tissues [9]. However, CHX lacks the ability to dissolve tissue or effectively remove the smear layer. Consequently, the residual smear layer may act as a barrier, reducing the contact time between the irrigant and the dentine [10].

In recent years, lasers have gained popularity in dentistry due to their broad range of applications, one of which is dental cavity disinfection. In addition to effectively penetrating and eliminating bacteria, lasers can also seal dentinal tubules, thereby preventing potential pathways for bacterial reinfection. Furthermore, lasers have the ability to remove the smear layer, consequently increasing the bonding strength between restorative material and dentine [11].

Among the lasers widely used as cavity disinfectants are the Diode 980 nm and Erbium YAG 2840 nm lasers. Diode lasers offer the advantage of effectively removing the smear layer and inducing melting of the dentine surface, resulting in partial to complete obliteration of dentinal tubules [2].

Low-energy Er:YAG laser treatment modifies the dentine surface by efficiently removing debris and exposing open dentinal tubules. Research indicates that the heat generated by the Er:YAG laser can neutralise free radicals and alter the dentine, resulting in a surface more favourable for bonding [12].

To the best of our knowledge, no study has compared the effects of chlorhexidine, diode laser 980 nm, and Er:YAG laser on dentine morphology and mineral content when used for dentine disinfection. Therefore, this study was conducted to assess the morphological changes and mineral content of dentine using scanning electron microscopy (SEM) and energy-dispersive X-ray spectroscopy (EDS), as well as to evaluate microleakage of composite resin restorations using SEM.

The null hypothesis stated that there would be no significant differences among chlorhexidine, Er:YAG laser, and diode laser (980 nm) when used for dental cavity disinfection with respect to: (1) the mineral content of dentine, as quantitatively assessed; and (2) the degree of microleakage at the tooth–restoration interface at both the occlusal and gingival margins.

## Materials and Methods

### Study design

This in vitro experimental study adhered to the CRIS Guidelines (Checklist for Reporting in Vitro Studies) published in 2014 to ensure methodological transparency and research quality [13]. A total of 40 freshly extracted human primary molars were used. To minimize structural variability, extractions were carried out within the expected exfoliation age range, ensuring that at least half of the root structure was still intact.

### Sample size calculation

Sample size was determined based on a previous study by Jamel and Taher (2024), which evaluated the antibacterial efficacy of a 940 nm diode laser on *Streptococcus mutans* and other cariogenic bacteria. Their study reported a mean ± SD of colony-forming units (CFU) as 34 ± 5.8 in Group I and 25 ± 3.6 in Group II, yielding an effect size of 1.86. Using a power of 0.9 and a significance level (α) of 0.05, the minimum required sample size was calculated to be eight per group. To account for a potential 20% dropout rate, this was increased to ten per group. The calculation was performed using a t-test in G*Power software (version 3.1.4.9) [14].

### Sample selection and randomizaton 

Randomisation and allocation were performed by labelling all 40 extracted teeth numerically (1–40) and storing them in a sterile, sealed, opaque container. Random allocation into four equal groups (*n* = 10) was carried out using a computer-generated random sequence from www.random.org on 12 November 2024, applying a 1:1 allocation ratio. A double-blind design was adopted: both the SEM and EDS examiners, as well as the statistician responsible for data analysis, were blinded to the group assignments.

The inclusion criteria required sound, freshly extracted teeth within the expected exfoliation age range, each with at least half of the root structure intact. Teeth exhibiting cracks, restorations, deep caries, pulpal involvement, structural anomalies, or previous dental treatment were excluded.

Teeth were collected from the outpatient paediatric dentistry clinic of the National Research Centre (NRC). Informed parental consent was obtained prior to extraction, and parents were briefed regarding the study involving their child’s extracted teeth. Following extraction, teeth were rinsed with tap water, residual soft tissues were carefully removed using a scalpel, and the crowns were gently cleaned with a soft-bristled brush (Sulcus, Oral-B, Mexico). Prior to analysis, specimens were stored in deionised water at 4 °C [15]. The structural integrity of all samples was initially assessed through visual inspection, followed by examination under a stereoscope (Olympus Optical Co., Ltd., Japan).

### Cavity preparation

Standardised Class V cavities were prepared on the buccal surfaces of each tooth using a high-speed handpiece equipped with a diamond bur (Horico Diament, Germany) under continuous water cooling. The cavity dimensions were fixed at 3 mm (mesiodistal) × 2 mm (occlusogingival) × 1.5 mm (depth), with the occlusal margin positioned 1 mm above the cementoenamel junction. Measurements were verified using a digital calliper. A new bur was used after every five cavities [16]. The teeth were sterilised by autoclaving at 121 °C for 15 minutes and stored individually in sterile, sealed test tubes [17].

### Experimental grouping

Teeth were randomly assigned to four groups:

- Group 1 (Negative Control): No disinfection was applied.
- Group 2 (Positive Control): Disinfection was performed using a 2% chlorhexidine gluconate solution (Grace for Dental Industries, Egypt), applied at a flow rate of 10 mL/min for 60 seconds. Following application, the cavity was rinsed with sterile saline and air-dried to remove any residual solution [18].
- Group 3 (Diode Laser): A 980 nm diode laser (LASOTRONIX, Poland) was used at a power of 1 Watt in continuous mode. The cavity surface was irradiated for a total of 60 seconds, divided into two 30-second cycles. An 8 mm tip was positioned 1 mm from the cavity in a perpendicular orientation and moved in a scanning motion to ensure even coverage. The spot size was 8 mm [9].
- Group 4 (Er:YAG Laser): Treatment was carried out using a 2940 nm Er:YAG laser (Fotona: AT Fidelis Ljubljana, Slovenia) with settings of 120 mJ pulse energy, 1.2 Watt power output, 10 Hz frequency, water and air levels set to 4, and SP (short pulse) mode with a pulse duration of 300 microseconds. Irradiation was delivered using an R02 tipless handpiece with a spot size of 0.9 mm for a total of 60 seconds, divided into two 30-second cycles [12].

The power of the Er:YAG laser was selected based on a pilot study conducted prior to the main study. The energy range between 50 and 150 mJ is considered safe regarding dentine morphology and mineralisation. The pilot study tested 50, 100, 120, and 150 mJ. Both 120 mJ and 150 mJ energies demonstrated the highest antibacterial efficacy against *Streptococcus mutans* with no statistically significant difference between them. Therefore, the energy setting of 120 mJ was chosen for the Er:YAG laser.

### Assessment of the effects of different treatment modalities

#### Assessment of morphological changes of dentine via Scanning Electron Microscopy (SEM) Analysis

Imaging was performed at baseline to capture the normal dentine morphology, and subsequently after the application of each treatment modality to examine the morphological changes in each group. Samples were examined using a Quanta 250 FEG scanning electron microscope (SEM) equipped with an energy-dispersive X-ray (EDS) unit. Imaging was conducted at 30 kV with magnifications of ×2000 and ×6000. The resolution of the electron gun reached 1 nm.

#### Assessment of mineral content changes of dentine via Energy Dispersive X-ray (EDS)

Samples were analysed using the EDS device attached to the Quanta 250 FEG SEM to determine the elemental and quantitative composition of dentine.

### Restoration Procedures

Following disinfection and examination using scanning electron microscopy (SEM) and energy-dispersive X-ray spectroscopy (EDS), the cavities were etched with Prime Dent Etch (USA) for 20 seconds, rinsed with water for 10 seconds, and gently air-dried using an air syringe. Subsequently, a universal bonding agent (Prime and Bond Universal, Dentsply Sirona, Germany) was applied with a microbrush for 20 seconds, air-thinned for 5 seconds, and light-cured for 10 seconds. The restoration was carried out using Spectra ST HV Composite (Dentsply Sirona, Germany), applied in 2 mm increments, each of which was light-cured for 20 seconds [19].

### Thermocycling process

The samples were immersed in distilled water at 37 °C for 24 hours, followed by thermocycling with 500 cycles. Each cycle consisted of 30 seconds at 55 °C, a 10-second pause at ambient temperature, 30 seconds at 5 °C, and an 8-second transition back to the initial condition, totalling 78 seconds per cycle. Thermocycling was performed using a TC-300 thermocycler (Vafai Factory) [20].

### Microleakage testing

#### Sample preparation for dye penetration

##### Microleakage Assessment via Scanning Electron Microscopy (SEM) Analysis

Following thermocycling, all specimens were thoroughly dried, and their apical regions sealed with sticky wax to prevent dye penetration. Two consecutive layers of nail varnish were applied, leaving an uncoated gingival margin of approximately 1 mm and maintaining a 2 mm clearance from the tooth–restoration interface, thereby ensuring dye infiltration occurred exclusively at the interface region [9]. The specimens were then immersed in 2% methylene blue solution (Sparks, USA) for 24 hours at room temperature. After immersion, the teeth were rinsed, dried, and sectioned longitudinally along the buccolingual axis using a microtome (MTI Corporation, Richmond, CA) [21].

Assessment of linear dye penetration at the tooth–restoration interface, both occlusal and gingival, was performed using the Quanta 250 FEG SEM to evaluate the degree of microleakage in micrometres.

### Statistical analysis

Mean and standard deviation values were calculated for each group under all test conditions. The data distribution was assessed for normality using the Kolmogorov–Smirnov and Shapiro–Wilk tests, confirming a parametric (normal) distribution. For comparisons involving more than two independent groups, one-way analysis of variance (ANOVA) was applied, followed by Tukey’s post hoc test for pairwise comparisons. The paired sample t-test was employed to analyse differences between two related groups. A significance level of *p* ≤ 0.05 was adopted for all statistical tests. Data analysis was performed using IBM® SPSS® Statistics software, version 20 (IBM Corp., Armonk, NY, USA).

### Ethics

The authors accept full responsibility for all aspects of the research, ensuring that any issues related to the accuracy or integrity of any component are thoroughly investigated and appropriately addressed. All procedures were conducted in full compliance with the ethical principles outlined in the Declaration of Helsinki. Informed consent was obtained from all participants prior to their involvement in the study. The experimental protocol was reviewed and approved by the Medical Research Ethics Committee of the National Research Centre (Approval no. 1234052022) on 7 April 2022.

## Results

### Morphological changes of dentine via SEM Analysis

**Fig. 1:**
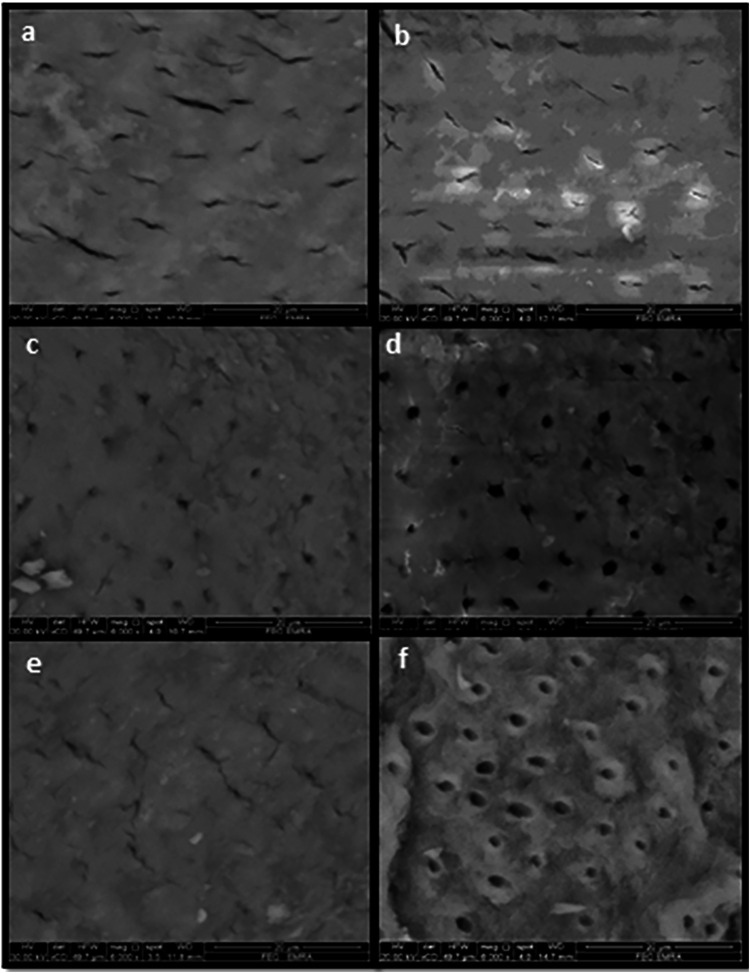
SEM Image of dentin before and after application of disinfectants. SEM image of dentine after cavity preparation and before application of various disinfectants showing partial occlusion of dentinal tubules with presence of a smear layer (**a**, **c**, **e**), after 2% CHO application, showing an apparent narrowing of dentinal tubules with precipitation of some particle with lack of removal of the smear layer (**b**), after diode laser application, showing an apparent widening of dentinal tubules with partial removal of the smear layer (**d**), after Er:YAG laser application, showing an obvious widening of dentinal tubules with removal of the smear layer (**f**).

SEM examination of the dentine surface after cavity preparation and prior to the application of disinfection modalities (baseline) revealed partial occlusion of dentinal tubules with the presence of a smear layer in all groups **(**Fig. 1a, c, e**)**. Following application of 2% chlorhexidine (CHX), SEM images showed apparent narrowing of dentinal tubules accompanied by precipitation of particulate matter, with no removal of the smear layer observed (Fig. 1b). After diode laser treatment, SEM images demonstrated apparent widening of the dentinal tubules and partial removal of the smear layer (Fig. 1d). In contrast, SEM images following Er:YAG laser application revealed marked widening of the dentinal tubules, complete removal of the smear layer, and melting of the dentine surface **(**Fig. 1f**)**

### Mineral content changes of dentine via EDS

#### A) Ca, P and Ca/P ratio of different groups

##### Calcium (Ca) change

as shown in Table 1 a statistically significant decrease in Ca content was observed when comparing pre- and post-treatment values across all groups (*p* < 0.001). The highest mean Ca value was recorded in the pre-treatment measurements, whereas the lowest values were detected post-treatment.

**Table 1:** The mean, standard deviation (SD) values of Ca, P, and Ca/P ratio of different groups.

Variables	EDS											
	Ca				*P*				Ca/P ratio			
	Pre		Post		Pre		Post		Pre		Post	
	Mean	SD	Mean	SD	Mean	SD	Mean	SD	Mean	SD	Mean	SD
**CHX**	58.89	1.18	42.90	0.82	20.80	0.12	17.45	0.13	2.83	0.06	2.46	0.06
**Diode laser**	57.58	1.12	43.17	0.49	20.49	0.39	17.58	0.44	2.82	0.08	2.47	0.07
**Er-YAG laser**	59.52	0.42	52.95	0.33	20.71	0.23	19.93	0.22	2.88	0.03	2.66	0.03
***p-value***	**0.372 ns**		<0.001*		**0.719 ns**		<0.001*		**0.785 ns**		0.030*	

* Statistically significant at *p* < 0.05. The *p*-value represents the probability that the observed differences occurred by chance; lower *p*-values indicate stronger evidence against the null hypothesis.

ns Non-significant (*p* > 0.05).

When comparing groups, no statistically significant difference existed among CHX (58.89 ± 1.18), Diode laser (57.58 ±1.12), and Er:YAG laser (59.52 ± 0.42) in the pre-treatment Ca content (*p* = 0.372), indicating that baseline mineral levels were standardized. However, post-treatment, a statistically significant difference was detected among CHX (42.90 ± 0.82), Diode laser (43.17 ± 0.49), and Er:YAG laser (52.95 ± 0.33) groups (*p* < 0.001). Pairwise comparisons revealed that the Er:YAG laser group had significantly higher Ca content than both the CHX and Diode laser groups (*p* < 0.001 for each), while no significant difference was found between CHX and Diode laser (*p* = 0.941). Overall, the Er:YAG laser group showed the highest post-treatment Ca levels, and the CHX group showed the lowest.

##### II) P content changes:

As shown in Table 1, a statistically significant decrease in P content was found when comparing pre- and post-treatment values across all groups (*p* < 0.001). The highest mean values were recorded in the pre-treatment measurements, while the lowest values occurred after treatment.

When comparing groups, no statistically significant difference was observed in pre-treatment P content among the CHX (20.80 ± 0.12), Diode laser (20.49 ± 0.39), and Er:YAG laser (20.71 ± 0.23) groups (*p* = 0.719), indicating that baseline P levels were standardized. In contrast, post-treatment values showed a statistically significant difference among CHX (17.45 ± 0.13), Diode laser (17.58 ± 0.44), and Er:YAG laser (19.93 ± 0.22) groups (*p* < 0.001). Pairwise comparisons revealed that the Er:YAG laser group had significantly higher P content than both the CHX and Diode laser groups (*p* < 0.001 for each comparison), while no significant difference was found between CHX and Diode laser (*p* = 0.950). Overall, the Er:YAG laser group showed the highest post-treatment P content, and the CHX group the lowest.

##### III) Ca/P ratio changes:

As shown in Table 1, a statistically significant difference was observed between the pre- and post-treatment Ca/P ratios across all groups (*p* < 0.001). The highest mean values were recorded before treatment, while the lowest values appeared after treatment.

Comparison of groups demonstrated that **pre-treatment** Ca/P ratios did not differ significantly among CHX (2.83 ± 0.06), Diode laser (2.82 ± 0.08), and Er:YAG laser (2.88 ± 0.03) groups (*p* = 0.785), indicating that the baseline Ca/P ratio was standardized. However, **post-treatment** values showed a statistically significant difference among CHX (2.46 ± 0.06), Diode laser (2.47 ± 0.07), and Er:YAG laser (2.66 ± 0.03) groups (*p* = 0.030). Pairwise comparisons revealed that the Er:YAG laser group had significantly higher Ca/P ratios than both CHX (*p* = 0.040) and Diode laser (*p* = 0.043) groups, while no significant difference was found between CHX and Diode laser (*p* = 0.996). Overall, the highest post-treatment Ca/P ratio was observed in the Er:YAG laser group, and the lowest in the CHX group.

#### B) Percentage of change of Ca, P and Ca/P of different groups

##### i. Relation between different groups according to Ca percentage of change:

As shown in Table 2, a statistically significant difference was observed among the CHX (27.12% ± 0.69), Diode laser (24.92% ± 0.82), and Er:YAG laser (11.02% ± 0.45) groups (*p* < 0.001). Pairwise comparisons demonstrated that the Er:YAG laser group differed significantly from both the CHX and Diode laser groups (*p* < 0.001 for each comparison), while no statistically significant difference was found between the CHX and Diode laser groups (*p* = 0.073). The CHX group exhibited the highest mean percentage change

**Table 2:** The mean, standard deviation (SD) values of the percentage of change of Ca, P, and Ca/P of different groups.

Variables	Percentage of change					
	Ca		*P*		Ca/P rati	
	Mean	SD	Mean	SD	Mean	SD
**CHX**	27.12%	0.69	16.09%	0.45	13.14%	0.63
**Diode Laser**	24.92%	0.82	14.29%	0.64	12.38%	0.94
**Er-YAG laser**	11.02%	0.45	3.76%	0.32	7.55%	0.34
***p-value***	<0.001*		<0.001*		<0.001*	

^*^ Statistically significant at *p* < 0.05. The *p*-value indicates the probability that the observed differences occurred by chance.

##### ii. Relation between different groups according to P percentage of change:

As shown in Table 2, a statistically significant difference was observed among the CHX (16.09% ± 0.45), Diode laser (14.29% ± 0.64), and Er:YAG laser (3.76% ± 0.32) groups (*p* < 0.001). Pairwise comparisons revealed that the Er:YAG laser group differed significantly from both the CHX and Diode laser groups (*p* < 0.001 for each), while no statistically significant difference was found between the CHX and Diode laser groups (*p* = 0.050). The CHX group showed the highest mean percentage change, whereas the Er:YAG laser group recorded the lowest.

##### iii. Relation between different groups according to Ca/ P ratio percentage of change:

As shown in Table 2, a statistically significant difference was observed among the CHX (13.14% ± 0.63), Diode laser (12.38% ± 0.94), and Er:YAG laser (7.55% ± 0.34) groups (*p* < 0.001). Pairwise comparisons indicated that the Er:YAG laser group differed significantly from both the CHX and Diode laser groups (*p* < 0.001 for each), while no statistically significant difference was found between the CHX and Diode laser groups (*p* = 0.712). The CHX group exhibited the highest mean value, whereas the lowest mean value was observed in the Er:YAG laser group.

### Microleakage assessment via SEM

Microleakage results are summarized in Table 3.

**Table 3:** The mean, standard deviation (SD) values of microleakage of different groups.

Variables	Microleakage					
	Occlusal		Gingival		Average	
	Mean	SD	Mean	SD	Mean	SD
**Control**	7.45	0.35	15.17	0.25	11.31	0.11
**CHX**	7.44	1.14	6.60	1.10	7.02	0.02
**Diode laser**	4.96	1.30	5.69	0.27	5.33	0.79
**Er-YAG laser**	3.52	0.51	1.34	0.24	2.44	0.22
***p-value***	<0.001*		< 0.001*		< 0.001*	

^*^ Statistically significant at *p* < 0.05. The p-value indicates the probability that the observed differences occurred by chance.

#### At occlusal margin of the cavity

A Statistically significant difference was observed among the Control (7.45 ± 0.35), CHX (7.44 ± 1.14), Diode laser (4.96 ± 1.30) and Er-YAG laser groups (3.52 ± 0.51) (*p* < 0.001). Comparisons revealed significant differences between the Control group and both of Er-YAG laser and Diode laser groups (*p* < 0.001), meanwhile no statistically significant difference was observed between the Control and CHX groups (*p* = 0.999).

A statistically significant difference was observed between the CHX group and each of Er-YAG laser and Diode laser groups (*p* < 0.001).

Also, a statistically significant difference was observed between the Er-YAG laser and Diode laser groups (*p* = 0.006).

The highest mean value was recorded in the Control, group whereas the lowest mean value was observed in the Er-YAG laser group.

#### At gingival margin of the cavity

A statistically significant difference was observed among the Control (15.17 ± 0.25), CHX (6.60 ± 1.10), Diode laser (5.69 ± 0.27), and Er-YAG laser groups (1.34 ± 0.24) (*p* < 0.001). Comparisons revealed significant differences between the Control group and each of CHX, Er-YAG laser, and Diode laser groups (*p* < 0.001),

A statistically significant difference was observed detected the CHX group and each of the Er-YAG laser and Diode laser groups (*p* < 0.001) and (*p* = 0.008).

Also, a statistically significant difference was observed between the Er-YAG laser and the Diode laser groups (*p* < 0.001).

The highest mean value was recorded in the Control group, whereas the lowest mean value was observed in the Er-YAG laser group.

#### Average of microleakage both occlusally and gingivally

A Statistically significant difference was observed among the Control (11.31 ± 0.11), CHX (7.02 ± 0.02), Diode laser (5.33 ± 0.79), and Er-YAG laser groups (2.44 ± 0.22) (*p* < 0.001).

Comparisons revealed significant differences between the Control group and each of the CHX, Er-YAG laser, and Diode laser groups (*p* < 0.001).

A statistically significant difference was detected between the CHX group and each of the Er-YAG laser and Diode laser groups (*p* < 0.001).

Also, a statistically significant difference was found between the Er-YAG laser and the Diode laser groups (*p* < 0.001).

The highest mean value was recorded in the Control group, whereas the lowest mean value was observed in the Er-YAG laser group.

## Discussion

Contemporary caries removal techniques may not reliably eliminate all microorganisms from the prepared cavity. Several studies have demonstrated that bacteria can persist within the dentine even after the application of caries detector dyes. Furthermore, research indicates that fermentative microorganisms may survive beneath restorations lacking antiseptic properties for up to 139 days [16].

These findings underscore the importance of incorporating cavity disinfection as an adjunct to conventional caries removal procedures to eliminate residual microbial contamination. This approach may help to reduce the risk of secondary caries, postoperative pulp sensitivity, and pulpal inflammation prior to definitive restoration.

In our study, three disinfection methods—2% chlorhexidine (CHX), 980 nm diode laser, and Er:YAG laser—were compared with a control group in terms of their effects on dentine morphology, mineral content, and microleakage. The results revealed that all disinfection techniques produced significant alterations in the dentine surface when compared with untreated controls, with the Er:YAG laser demonstrating the most favourable outcomes across all evaluated parameters.

Chlorhexidine was employed in this study due to its well-established antimicrobial efficacy, particularly its inhibition of streptococcal species, and its substantivity on dentine surfaces [8]. A 2% aqueous solution was selected, as it is widely used in clinical and research settings and is considered biocompatible with an acceptable toxicological profile [22]. However, despite its antimicrobial benefits, our findings align with those of Haralur et al. and Kimyai et al., who reported that CHX adversely affects dentine by reducing calcium content and hardness [5], and by compromising the bonding and sealing of adhesive restorations to dentine, potentially increasing microleakage [3].

Lasers have been widely employed as effective alternatives to chemical disinfectants due to their potent antibacterial effects and favourable impact on dentine, such as smear layer removal and improved bonding potential [4, 12, 23, 24]. The interaction between laser energy and dental tissues is strongly influenced by the laser’s wavelength and power density [4]. Diode lasers are available in various wavelengths, including 810–830 nm, 940 nm, 980 nm, and 1064 nm. In the present study, the 980 nm diode laser was selected for its proven antibacterial efficacy and its capacity to partially remove the smear layer [23].

Low-energy Er:YAG laser treatment modifies the dentine surface by efficiently removing debris and exposing open dentinal tubules. Previous research has shown that the heat generated by Er:YAG lasers can neutralise free radicals and alter the dentine surface, thereby enhancing its suitability for bonding procedures [7]. Operating at a wavelength of 2.94 µm, the Er:YAG laser has demonstrated bactericidal effects against Streptococcus mutans. In this study, a low energy output of 1.2 watts was used to minimise thermal damage to dentine and prevent potential reductions in dentine hardness. This parameter selection is supported by Du et al., who found that power settings between 1 and 1.5 watts effectively reduced S. mutans levels without compromising the structural integrity of dentine [25].

Scanning electron microscopy (SEM) was utilised in this study to assess ultrastructural changes on the dentine surface following treatment application, given its capacity for both qualitative and quantitative morphological evaluation [26]. SEM analysis revealed that dentine treated with 2% chlorhexidine exhibited irregular dentinal tubules with partial occlusion and residual smear layer presence. This aligns with Lapinska et al. [27], who observed irregular deposits distributed across dentine surfaces, and Siwnata et al. [28], who reported incomplete smear layer removal by 2% chlorhexidine gluconate, resulting in smear plugs that impeded full dentinal tubule exposure.

Dentine treated with a 980 nm diode laser at 1 W demonstrated an irregular surface with partial smear layer removal and apparent dentinal tubule opening. These results concur with Behniafar et al. [29], who found no significant pulpal temperature increase or dentine structural damage at this power level. Conversely, Jhingan et al. [23] and El Tayeba et al. [4] reported total smear layer removal with morphological changes such as dentine melting, likely due to their use of higher laser powers (2 W and 1.5 W, respectively). Abdou et al. [26] similarly noted effective smear removal at 1.5 W but cautioned about potential pulp damage, supported by Jaine et al.’s findings that 1 W diode laser is safer than 2 W, despite comparable antibacterial effects [30].

Our findings show that Er:YAG laser treatment resulted in marked widening of dentinal tubules and complete smear layer removal, consistent with Vieira et al. and Wang et al. [16, 24], who reported enhanced bonding due to the retentive surface pattern created. However, Burlat et al. [31] observed surface cracks and dentine disintegration at Er:YAG powers between 250–300 mJ, likely attributable to excessive laser energy, a conclusion supported by Wanderley et al. [32].

Overall, the SEM results support the hypothesis that different cavity disinfection methods distinctly affect dentine morphology, with Er:YAG laser showing enhanced smear layer removal and surface conditioning compared to diode laser and chlorhexidine, thereby potentially improving restorative bonding and clinical outcomes.

Calcium (Ca) and phosphorus (P), constituents of hydroxyapatite crystals, represent the primary inorganic components of dental hard tissues. Variations in the Ca/P ratio can disrupt the natural balance between organic and inorganic components, potentially altering dentine’s structural properties, including permeability and solubility [33].

In this study, energy-dispersive X-ray spectroscopy

(EDS) was employed to assess changes in the mineral content of dentine following various disinfection modalities, selected for its accuracy and sensitivity in mineral quantification [26]. All experimental groups demonstrated lower Ca/P weight ratios compared to their untreated controls.

The observed reduction in calcium and phosphorus levels after chlorhexidine (CHX) application aligns with findings reported by Haralur et al. and Kimyai et al. [5]. This decrease can be attributed to the cationic nature of CHX, which facilitates binding to anionic molecules such as phosphate groups in hydroxyapatite, leading to the displacement and release of calcium ions from dentine. These findings suggest that CHX contributes to calcium ion removal via its interaction with phosphate [3, 5, 34].

Regarding diode laser treatment, statistical analysis revealed a significant difference in calcium and phosphorus content before and after treatment, consistent with Azmy et al., who reported that diode laser surface treatment induces measurable changes in the mineral composition of radicular dentine [35].

Conversely, Abdou et al. reported no chemical alterations in dentine components following diode laser application. This discrepancy may be explained by their use of distilled water as an irrigant immediately prior to 980 nm diode laser irradiation, which could absorb the heat generated by the laser’s photothermal effect, thereby preserving dentine’s inorganic content [26].

Er:YAG laser treatment resulted in the smallest percentage change in calcium and phosphorus content. These findings concur with Soares et al. [33], who observed minimal alterations in elemental composition and a slight reduction in the Ca/P ratio following Er:YAG laser irradiation at 100 mJ. Conversely, Moosavi [36] reported increased dentine microhardness and calcium ion content post Er:YAG irradiation; however, these differences were not statistically significant compared to controls.

Microleakage is defined as the infiltration of oral fluids, molecules, bacteria, and ions at the interface between the cavity walls and the restorative material. Preventing microleakage is essential for ensuring the longevity and clinical success of dental restorations [11]. An ideal cavity disinfectant should therefore possess effective antimicrobial properties while preserving the sealing integrity of restorative materials. Any compromise to this seal may result in marginal leakage, allowing the ingress of bacteria and fluids, which can negatively affect restoration durability [14].

Microleakage can be assessed using several methods, among which the dye penetration technique—used in the present study—is one of the most widely adopted in recent research. This method is favoured due to its practicality: dye solutions are easily obtainable, the technique avoids the use of reactive chemicals or radiation, and it is reproducible and straightforward to implement [29].

Thermal cycling is commonly employed in in vitro studies to simulate the clinical ageing of dental restorations. In this study, all specimens were subjected to 5000 thermal cycles between 5 °C and 55 °C prior to microleakage assessment [15].

The group treated with chlorhexidine demonstrated reduced microleakage at the tooth–restoration interface compared to the control group. These findings are consistent with those reported by Ramezanian et al. [37], who found that chlorhexidine significantly reduced microleakage both immediately post-restoration and over time. Similarly, Satpute’s review highlighted the role of chlorhexidine in enhancing the longevity of restorative materials [36].

However, these outcomes differ from those of Mutluay et al., who reported no significant effect of chlorhexidine on microleakage compared to controls. This discrepancy may be attributed to variations in experimental protocols, such as the duration of chlorhexidine application and the restorative material used; their study employed a giomer-based material, whereas composite resin was used in the current study [2].

In the present study, the 980 nm diode laser group exhibited lower microleakage values than both the control and chlorhexidine groups. These findings are consistent with El Mansy et al., who reported that using a 980 nm diode laser at the same power and exposure time led to the lowest statistically significant microleakage values compared with the CHX and control groups. Conversely, Ipek et al. observed no significant improvement in microleakage following diode laser treatment. Such variations may stem from differences in laser parameters, application time, or the type of restorative material used across studies [9].

Regarding the Er:YAG laser, our results indicated that it achieved the lowest microleakage levels among all tested groups. This is supported by a study by Emilie Luong and Amir Shayegan, which demonstrated that Er:YAG laser conditioning of enamel and dentine beneath resin composite effectively reduced microleakage [38]. Similarly, Sharafeddin and Tabrizi found that Er:YAG laser treatment resulted in less microleakage at both occlusal and gingival margins when compared to CO₂ laser application [7]. Ipek Aslan et al. also reported that the Er,Cr:YSGG laser significantly outperformed the diode laser and chlorhexidine in reducing nanoleakage [17].

To the best of our knowledge, no previous study has directly compared the efficacy of Er:YAG laser with diode laser or chlorhexidine for cavity disinfection, nor has such a comparison been made regarding their respective effects on dentine morphology.

According to the results of this study, the null hypothesis was rejected since statistically significant differences were observed not only between the control and the treated groups, but also among the different disinfection methods tested. In particular, Er:YAG laser demonstrated enhanced outcomes compared with chlorhexidine and diode laser.

## Conclusion

Within the limitations of this study, it can be concluded that the Erbium: YAG laser demonstrated the most favourable outcomes in terms of preserving and enhancing both the morphological and chemical composition of dentin. Moreover, it resulted in the lowest microleakage values among all tested groups, indicating enhanced sealing ability and potential for long-term restoration success.

Both the diode laser and 2% chlorhexidine (CHX) showed beneficial effects compared to the control, with no significant difference between them.

CHX exhibited the highest microleakage values, suggesting a less favourable impact on the marginal integrity of restorations.

### Limitations

Future investigations should explore using disinfecting agents with application times shorter than 60 seconds. Because mineral content and microleakage were assessed only immediately after treatment, the long-term stability and durability of the restorations remain unknown; thus, studies with extended follow-up periods are needed to provide more comprehensive evidence. Additionally, future research should incorporate clinically relevant conditions—such as pulpal pressure simulation, natural biofilms, and in vivo validation—to improve the practical applicability of the findings.

### Recommendation

More future studies should be done using modern injectable glass ionomer materials

## Data Availability

The datasets generated and/or analyzed during the present study are available from the corresponding author upon reasonable request. All measures were taken to ensure the protection of participants’ privacy.
